# Impact of Helmet-Wearing Policy on E-Bike Safety Riding Behavior: A Bivariate Ordered Probit Analysis in Ningbo, China

**DOI:** 10.3390/ijerph19052830

**Published:** 2022-02-28

**Authors:** Jibiao Zhou, Tao Zheng, Sheng Dong, Xinhua Mao, Changxi Ma

**Affiliations:** 1 College of Transportation Engineering, Tongji University, Shanghai 200082, China; zhoujb2014@nbut.edu.cn; 2 School of Civil and Transportation Engineering, Ningbo University of Technology, Ningbo 315211, China; zheng_tao2018@163.com; 3 College of Transportation Engineering, Chang’an University, Xi’an 710064, China; maoxinhua@chd.edu.cn; 4 School of Traffic and Transportation, Lanzhou Jiaotong University, Lanzhou 730070, China; machangxi@mail.lzjtu.cn

**Keywords:** electric bicycle, BOP model, helmet policy, cycling behavior, interventions

## Abstract

At present, Chinese authorities are launching a campaign to convince riders of electric bicycles (e-bikes) and scooters to wear helmets. To explore the effectiveness of this new helmet policy on e-bike cycling behavior and improve existing e-bike management, this study investigates the related statistical distribution characteristics, such as demographic information, travel information, cycling behavior information and riders’ subjective attitude information. The behavioral data of 1048 e-bike riders related to helmet policy were collected by a questionnaire survey in Ningbo, China. A bivariate ordered probit (BOP) model was employed to account for the unobserved heterogeneity. The marginal effects of contributory factors were calculated to quantify their impacts, and the results show that the BOP model can explain the common unobserved features in the helmet policy and cycling behavior of e-bike riders, and that good safety habits stem from long-term safety education and training. The BOP model results show that whether wearing a helmet, using an e-bike after 19:00, and sunny days are factors that affect the helmet wearing rate. Helmet wearing, evenings during rush hour, and picking up children are some of the factors that affect e-bike accident rates. Furthermore, there is a remarkable negative correlation between the helmet wearing rate and e-bike accident rate. Based on these results, some interventions are discussed to increase the helmet usage of e-bike riders in Ningbo, China.

## 1. Introduction

As an emerging transport tool, electric bicycles (e-bikes) have been widely used due to their low cost and convenient travel characteristics [1,2,3,4,5,6,7]. By the end of 2018, the number of e-bikes in China had reached 250 million, with 69 e-bikes per 100 urban households, an increase of 47.44% from 2013 [7,8]. Despite the obvious advantages of e-bikes, their rapid growth has raised a number of safety issues. Statistics show that the total number of e-bike accidents in China was 40,400 in 2013 and reached 56,200 in 2017, an increase of 39.1% year on year, with an average annual growth rate of 8.6%. The number of casualties caused by e-bike accidents has increased, and the China Statistical Yearbook (National Bureau of Statistics of China 2017) [9] also shows that the number of e-bike fatalities was 733 in 2011 and reached 1305 in 2016, an increase of 78.02% in a five-year period. The number of e-bike injuries was 8532 in 2011 and reached 16,944 in 2016, an increase of 98.59%. Hence, large and medium-sized cities in China, such as Guangzhou, Shenzhen and Wenzhou, have issued policies to ban or restrict the use of e-bikes [10]. In addition, in May 2020, Jiangsu and Zhejiang Provinces issued “regulations on electric bicycles”, which explicitly require riders to wear helmets. Any individual who fails to wear a helmet in accordance with these regulations will be warned or fined ¥20 ($3.03) to ¥50 ($7.57) by the traffic administrative department.

Numerous studies [11,12,13,14,15,16,17,18] have shown that the failure to properly use seat belts and safety helmets is one of the major causes of road traffic accidents. In 2018, the number of e-bike rider fatalities in Ningbo [19] was 387, accounting for 44.79% of the total number of accident fatalities. Among them, deaths caused by head injuries accounted for 88.89% of all e-bike deaths. The data indicate that e-bike riders account for a high proportion of fatalities in road traffic accidents, and that using e-bikes without a helmet is a major cause of casualties. Therefore, it is necessary to strengthen helmet-wearing management.

Wearing a safety helmet can effectively reduce the risk of head injury, which has also been proven by a large number of previous studies [20,21,22], such as those using laboratory tests, real crash data tests, and case–control study methods. A study by Dorschet al. (1987) [23] demonstrated the efficacy of bicycle helmets in real crashes. It was found that the risk of death from head injury was considerably lower for helmeted bicyclists than unhelmet bicyclists. Subsequently, Thompson et al. (1996) [24] examined the effectiveness of bicycle helmets in preventing and protecting against head injuries. The results showed that bicycle helmets, regardless of types, provide protection to cyclists of all ages involved in crashes, including those involving motor vehicles. Recent studies have shown that wearing a safety helmet can reduce the risk of head injury [25,26,27]. These studies indicated that safety helmets provide obvious protection against head injuries. Consequently, interventions to decrease head injury should focus on increasing the helmet wearing rate.

To this end, our study aims to explore the effect of the new helmet policy on the compliance of e-bike riders. Our work makes the following three contributions. (a) We examine the contributing factors affecting crashes involving electric bicycles and helmet-wearing rates at a disaggregated level; (b) we integrate a complete set of original covariates, including demographic information, travel information, cycling behavior information and riders’ subjective attitude information, to examine the contributing factors related to crashes involving electric bicycles and helmet usage rates; and (c) we employ a robust statistical approach, i.e., a bivariate ordered probit (BOP) model, to explore the unobserved heterogeneity among observations. We also innovatively use marginal effects to quantify the impact of unobserved factors on crashes involving e-bikes and helmet usage rates.

The remainder of this paper is organized as follows. Section 2 reviews the existing studies on helmet usage rates, helmet effectiveness, and helmet laws and regulations in China. Section 3 presents a data collection survey and the corresponding statistical methods using a case study. Section 4 illustrates the survey results, model results, and policy-related measures. Conclusions and future work are discussed in Section 5.

## 2. Literature Review

### 2.1. Helmet Types and Helmet Usage Rates

**Figure 1:**
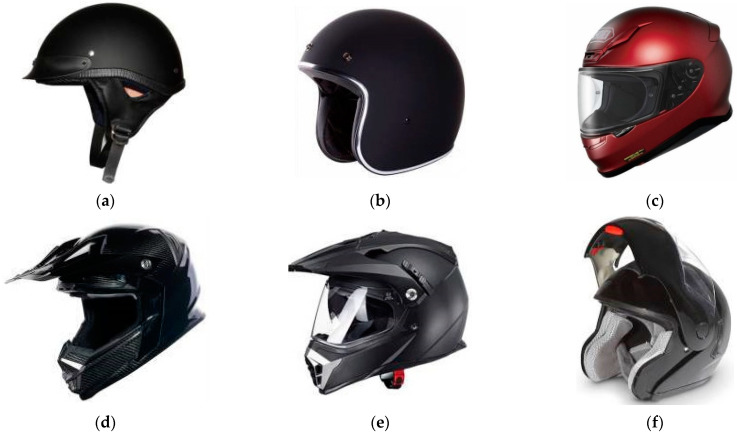
Types of helmet used by e-bike riders. (**a**) Half helmet, (**b**) three-quarters helmet, (**c**) full-face helmet, (**d**) off-road helmet, (**e**) tour-cross helmet, (**f**) flip-up helmet.

Helmets for motorcycles and electric bicycles are divided into three categories according to their shapes: (a) half helmets, (b) three-quarter or 3/4 helmets, and (c) full-face helmets. Moreover, full-face helmets can be broken into three subcategories: off-road helmets, tour-cross helmets, and flip-up helmets, as shown in Figure 1d–f. Generally, although the convenience and comfort of full-face helmets are not as good as those of 3/4 and half helmets, when an accident occurs, they can provide better chin protection. A recent study by Tabary, M. et al. (2021) [28] found that full-face motorcycle helmets may provide better protection from head and facial injury. The advantages that come with full-face helmets are not provided by other styles of helmets; half helmets have better breathability and are light in weight, which is more suitable in summer but offers less protection.

A large number of previous studies have proven that helmet usage can help promote e-bike riders’ responsibility in terms of road traffic safety. An earlier statistical survey [29] from the U.S. Consumer Product Safety Commission (CPSC) found that the helmet usage rate increased from 18% in 1991 to 50% in 1998. A survey report [30] by the National Highway Traffic Safety Administration (NHTSA) of the U.S. Department of Transportation (DOT) investigated cyclist and pedestrian attitudes and behaviors in the U.S. in 2012, and found that at least half of cyclists wear helmets for some trips, while 35% of all cyclists wear helmets on all or most of their trips.

In the U.S., safety helmets have gradually begun to be accepted and used by cyclists and e-bike riders. In 1994, the usage rate of safety helmets was 62.5% and reached 70.8% in 2019 [31], which shows that the safety awareness of e-bike riders is high and that the acceptance of safety helmets is also increasing. Compared with the U.S., helmet usage rates in China are still low, and there is a serious imbalance in the proportion of users. According to a survey by iiMedia Research [32] in 2020, 70.11% of Chinese helmet users are male, and only 29.89% are female. Additionally, more than 30% of cyclists are reluctant to wear helmets due to sweltering weather, and nearly 20% of cyclists are reluctant to wear helmets due to the inconvenience of storing them. These results show that the safety awareness of Chinese e-bike riders is still weak.

### 2.2. Helmet Effectiveness and Collaborative Networks Analysis

**Figure 2:**
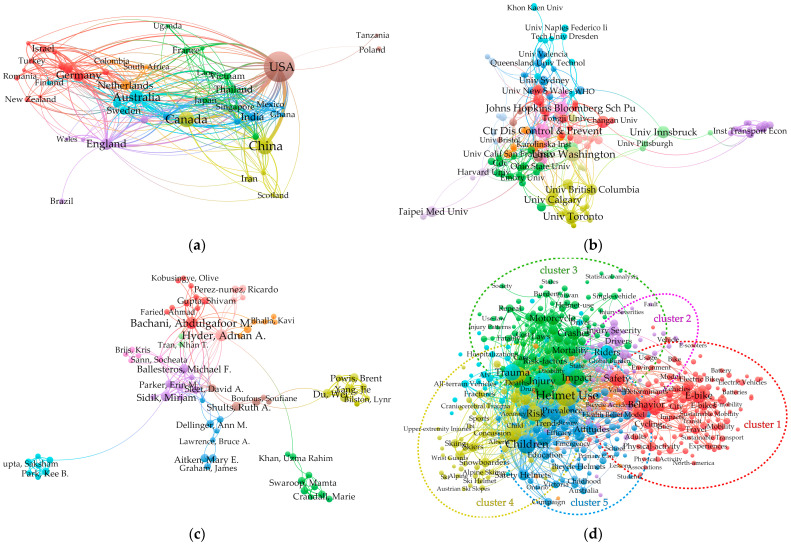
Collaborative networks analysis for helmet studies. (**a**) Collaborative networks between countries, (**b**) collaborative networks between research organizations, (**c**) co-authorship network, (**d**) keyword co-occurrence network.

The world report on road traffic injury prevention was prepared in 2004 by a joint survey reported by the world health organization (WHO) [33]. It was found that the correct wearing of motorcycle safety helmets can reduce the risk of death from traffic accidents by 40% and the risk of serious head injuries by 70%. For e-bike riders, wearing a safety helmet can also provide the same protection. However, in the early 1990s, the wearing rate of helmets was generally low, and there was controversy regarding whether they could effectively protect human life [34]. Since then, numerous studies [35,36,37,38] have shown that helmets can reduce the severity of head and spinal injuries, hospital stays, costs, and mortalities. Helmets have been shown to not only reduce the risk of head and brain injuries, but also provide substantial forehead and midface protection. A prospective case–control study by Thompson et al. [24,39] examined the effectiveness of bicycle helmets in preventing and protecting against head injuries. It was found that helmets provide a 66% to 88% reduction in the risk of severe head and brain injuries for bicyclists of all ages. Moreover, peer-reviewed studies [28,40] have confirmed that helmets have an important protective effect on the heads and faces of riders. It is estimated that 75% of bicycle-related fatalities among children could be prevented by wearing bicycle helmets. Meta-analysis results [27,41,42] have shown that the use of helmets can reduce cyclists’ head injuries by 48%, serious head injuries by 60%, brain injuries by 53%, and facial injuries by 23%. In this study, we applied the mapping knowledge domain (MKD) approach to perform a comprehensive and objective review of the helmet effectiveness of e-bikes studies, as shown in Figure 2. The Web of Science (WoS) Core Collection was selected as the data source in the MKD approach. We searched the literature from 1975 to 2022 using “e-bike”, “helmet effectiveness”, and “helmet” as keywords. After that, the VOSviewer was adopted as the bibliometric analysis software. VOSviewer was developed to offer a superior visualization of the mutation detection function, especially in co-occurrence network analysis.

Figure 2a maps the distribution of the main countries in the helmet effectiveness of e-bikes studies. The existing literature on helmet effectiveness of e-bikes comes from 50 countries. Figure 2a shows the number of studies issued from each country and the number of institutions represented. It can be seen that China and the United States have the largest number of articles, at 782 and 214, respectively, accounting for a total contribution rate of 48.87%. Figure 2b shows the cooperation among scientific research institutions by 2022, in which nodes of various colors show cooperative relationships, and the thickness of the link indicates the number of papers produced by the collaboration. In Figure 2c, each node represents an author, the size of the node represents the number of published articles, and the links between the nodes represent the cooperative relationship between the two authors. Keywords often involve subjects such as research interests, research fields, research objects, research topics, and research methods, which play important roles in revealing the research trends of studies. Keyword co-occurrence analysis (KCA) is a common research method in scientometrics [43]. Hence, it was used to generate the keyword co-occurrence network for helmet studies, as shown in Figure 2d. With reference to the characteristics and status of safety helmet studies, the keyword network can be divided into five clusters, namely, cluster 1 (red cluster), cluster 2 (purple cluster), cluster 3 (green cluster), cluster 4 (yellow cluster), and cluster 5 (blue cluster). The co-occurring keywords mainly include helmet use, children, injuries, risk, and impact, the proportions of which are 3.73%, 2.53%, 1.98%, 1.61% and 1.59%, respectively. As shown in Figure 2d, there is a significant correlation between the keywords in each cluster. (a) The red cluster focuses on riding behavior characteristics of e-bikes; (b) the purple cluster focuses on drivers, vehicle, and fault; (c) the green cluster focuses on trauma, mortality, and injuries; (d) the yellow cluster focuses on risk, helmet-use and physical damage; and (e) the blue cluster focuses on children, attitude, and emergency.

### 2.3. E-Bike Helmet Laws and Regulations in China

Road traffic collisions [44,45] are a global health problem and include injuries related to electric bicycles. In different countries, the fatality rate of injuries of e-bike riders is quite high, ranging from 5% to 90% [34,46]. In road traffic collisions, the probability of severe injury resulting from e-bike accidents was nearly two times that of regular bicycle accidents [45,46,47]. At the same time, previous studies [33,34,39,46,47] have also shown that the injury severity of e-bike riders is higher than that of motor vehicle drivers. Among these types of e-bike collisions, head injury is a common cause of severe morbidity and mortality. In addition, some studies have also shown that some factors, such as the road traffic characteristics [48], high-speed roads [49], weather, temperature [50], latent psychological factors [51], time pressure [52], traffic environment [53], and rider demographic factors, are also closely related to e-bike collisions. However, few studies have focused on e-bike helmet laws and regulations, particularly in China.

With the aim of enhancing the enforcement of wearing helmets for bicyclists and e-bike riders for some trips, helmet laws and related regulations have been supported by previous studies [19,54,55,56]. For instance, a study by Dong et al. (2021) [52] found that traffic enforcement has a more powerful inhibiting effect on the aggressive driving behaviors of food delivery riders. According to the new policy issued by China’s Ministry of Public Security (CMPS), fines will be handed out to e-bike riders who do not wear a helmet and car drivers who fail to fasten their seatbelts. These new regulations make helmet wearing mandatory for Chinese e-bike riders, and helmet safety promotions will be carried out nationwide. Since 1 July 2020, Zhejiang and Jiangsu Provinces have implemented “regulations on the safety management of electric bicycles” to warn and impose fines on those who do not wear safety helmets, aiming to further improve the safety and protection level of motorcycle and electric bicycle riders. For instance, motorcycle and e-bike riders who do not wear helmets could obtain a fine up to ¥200 ($30.28). This regulation has had a good demonstrative effect on helmet usage. This paper aims to extend the approach of previous studies with a focus on the effect of the new helmet policy on the compliance of e-bike riders, and examine the contributing factors related to crashes involving e-bikes and helmet usage rates.

## 3. Data and Methods

### 3.1. Data Collection

**Figure 3:**
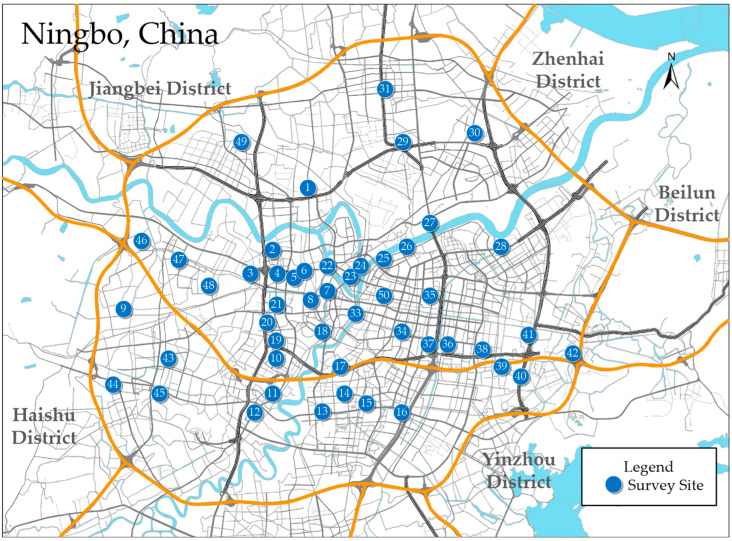
Survey site in Ningbo, China.

Questionnaire surveys have widely been used in traffic engineering research to collect information on helmet usage and crash involvement. In the current study, a questionnaire survey was conducted randomly by different survey teams in different locations. The survey was carried out by the Traffic Engineering Specialty Group of the School of Civil and Transportation Engineering of Ningbo University of Technology, and was conducted in five core areas of Ningbo: the Haishu, Jiangbei, Zhenhai, Yinzhou and Beilun Districts. The sample data of this research are true and valid. The detailed survey site in Ning as shown in Figure 3.

#### 3.1.1. Sampling Survey Method

A random sampling survey method was adopted to conduct a sample survey of e-bike users in Ningbo, China. The method used to estimate the sample size of the survey referred to the road accident rate *p* of e-bikes, which was 15.99%, in the study of Zhang et al. [47]. At the same time, a 95% confidence level was selected, assuming that the error δ was 0.25 and the confidence interval width (two-sided) *p*-value was 0.08. Based on the above parameters, a sample with a rejection rate of 10% was obtained from Ningbo, China; the final sample size *N* was 1048.

#### 3.1.2. Reliability and Validity Tests

Reliability and validity are the main criteria for testing whether a questionnaire is qualified, and are used to ensure that a questionnaire makes sense.

Reliability refers to questionnaire reliability. The data in the questionnaire were repeatedly tested. Each question involved a dimension. After investigation, all questions were used to measure the same content, and finally obtained consistent results, indicating that the questionnaire design was more reasonable. The higher the reliability coefficient, the more reliable the data source. Cronbach’s alpha is currently the most commonly used reliability coefficient. For general exploratory research, when Cronbach’s alpha is above 0.6, it is considered as having passed the test.

Validity refers to the questionnaire validity, which is measured using tools that identify the correctness of the questionnaire design and accurately reflect the validity of the data. The higher the validity coefficient, the better the questionnaire validity.

The reliability and validity of the questionnaire survey results were analyzed using SPSS 11.0. The analysis results show that the Cronbach’s alpha of the questionnaire is 0.663 > 0.6, which indicates that the scales of the variables have positive internal consistency and that the sample meets the requirements of a reasonable data structure, thus passing the reliability test. The validity analysis KMO = 0.644 > 0.5 indicates that the sample size is sufficient, and the sphere test coefficient *p* = 0.000 < 0.05 indicates that the sphere test has been passed; by referring to some similar studies [57,58,59], the research data can be used for extraction and analysis.

### 3.2. Questionnaire Design and Results

#### 3.2.1. Questionnaire Design

The questionnaire design is based on the results of an extensive review of the literature and pre-conducted group discussions. According to some studies, wearing motorcycle helmets can effectively provide head safety, and reduce crash involvement and the severity of such involvement. Before the investigation, pretests were conducted on 60 ordinary Ningbo e-bike riders to identify potential problems in the questionnaire and prevent deviations. Based on the feedback [26,27], the initial questionnaire was revised to make the questions clearer. In the questionnaire design, we referred to past surveys and studies on the wearing of safety helmets for e-bike riders, and to the information collected from previous studies, including gender, vehicle type, helmet-wearing condition, weather and other factors, as well as subjective safety awareness. At the same time, based on the helmet policy implemented by Ningbo, we also added factors such as cycling proficiency, policy awareness, and helmet protection capabilities. Although self-report surveys have limitations, especially in terms of some subjective descriptions, surveys provide the opportunity to supplement this analysis with detailed demographic data, which provides us with subjective attitude factors that have previously been ignored, such as “whether one is safe after wearing a helmet”, “cycling proficiency”, “road security”, “times of punishments when a helmet is not worn in cycling behavior”, “punishment degree” and “reasons for not wearing a helmet”, and their impact on helmet usage and crash involvement. In addition, cross-check questions were set up to filter out any self-reported bias. The questionnaire consisted of six parts, preceded by the following brief description.

From July to August 2019 (before the COVID-19 outbreak), students from the School of Transportation of Ningbo University of Technology (NBUT) were hired to take part in face-to-face surveys. The investigator asked respondents if they agreed to participate in the anonymous study, and participants verbally agreed to take part in the survey. The survey was conducted on weekdays and weekends to collect a wide range of interviewee types. Random sampling techniques were used to select e-bike riders. Investigators were asked to randomly select every five to ten people (those who were over 12 years old). After completing the questionnaire, each respondent received a ¥10 payment. A total of 1200 questionnaires were randomly assigned to respondents.

Finally, 1200 questionnaires were collected and checked for data selection. Questionnaires with the following issues were excluded: (a) respondents who had never experienced e-bike riding; (b) incomplete key information (for example, travel distance or travel purpose); (c) questionnaires in which respondents basically chose the highest or lowest Likert scale answer for all questions; (d) contradictory cases (such as young but retired respondents); and (e) cases that caused logic problems in the coding process. A total of 1048 samples were obtained after data selection.

#### 3.2.2. Questionnaire Results

Table 1, Table 2, Table 3 and Table 4 list all the items of the questionnaire survey and the basic characteristics of all respondents. Among them, the survey question concerning helmet usage after policy release was linked to the cycling behavior variable, and the survey question concerning crash involvement was linked to the travel information variable. Many explanatory variables were collected from the questionnaire to determine whether they affect helmet usage and crash involvement. As shown in the table, of the 1048 respondents, men accounted for 58.49%, and women accounted for 41.51%. The average age of respondents was 34.4 years. In terms of the educational level of the surveyed people, 7.44% had a junior high school degree or less, 28.15% had junior high school and high school degrees, and 54.58% had undergraduate degrees, which accounted for the largest portion of the sample. In addition, 9.83% of the surveyed people had master degrees.

As shown in Table 2, among the 1048 respondents, 32.44% travel within 1 to 3 km, and 27.39% travel within 3 to 5 km. As regards the travel time of the surveyed population, the morning peak and evening peak account for a large proportion of the sample. Additionally, people who travel for work and shopping account for 48.85% and 45.99%, respectively. As shown in Table 3, among the 1048 respondents, the number of people who never wore helmets before the release of the policy is the lowest, at 12.21%, and the frequency of wearing a helmet occasionally is the highest, 33.21%. After the release of the policy, the proportion of people who never wear helmets decreased to 4.01%, and the proportion of people who often wear helmets increased to 51.44% (18.80% before the release of the policy), indicating that the effect of the policy is obvious. As indicated in Table 4, among the 1048 respondents, 72.24% know about the policy, and 81.01% of riders think it is safer to wear helmets; 51.72% of riders are unwilling to wear helmets because they will cause discomfort; 48.76% of riders think that wearing helmets will affect their sight, and most of them are unwilling to wear helmets in hot weather.

The survey results show that 74.24% of users know about the helmet policy issued by Ningbo city, which shows that the policy has achieved strong publicity and has had a great influence. However, according to the survey results, only 51.44% of users said they would always wear helmets after the policy’s release, which means that e-bike users still do not pay enough attention to cycling safety issues. Another dataset also shows that only 31.20% of the surveyed users are fully aware that “the World Health Organization (WHO) points out that helmets can reduce the risk of death and injury”, while users who fully know the correct way of wearing safety helmets account for only 43.61%. This finding shows that the safety awareness of e-bike riders is weak.

According to the survey, the number of e-bike riders unwilling to wear safety helmets on rainy and hot days is 56.11% and 56.87%, respectively, which accounts for far more than those in other weather conditions. The reason for this may be that the weather on rainy days is muggy, which may cause discomfort to riders, such as breathing difficulties. Conversely, wearing safety helmets in hot weather can easily hinder bodily heat loss and affect the cycling experience of users. Regarding the reasons for the user being unwilling to wear safety helmets, discomfort and sight being blocked by safety helmets account for 51.72% and 48.76, respectively. This finding suggests that many users are dissatisfied with the comfort and convenience of safety helmets. Safety helmet manufacturers can improve the performance of safety helmets, such as the degree of air permeability, heat dissipation, and the convenient operation of safety helmets, according to the above factors, so that users are more willing to wear safety helmets.

### 3.3. Bivariate Ordered Probit Model

#### 3.3.1. BOP Model

The aim of this study is to explore the factors that affect helmet usage after policy release and crash involvement. Using discrete outcome modeling techniques, the dependent variables are composed of categorical variables. Considerations should be given to unobserved factors that affect helmet usage after policy release and crash involvement. The BOP model is used to identify the factors that affect both helmet usage after policy release and crash involvement. The BOP model is designed to simulate categorical dependent variables that can be determined simultaneously, and first defines the ordinal observation number of each observation as follows:

(1) $$\left\{ \begin{array}{l} y_{i,1}^{*}=\beta_{1}X_{i,1}+\varepsilon_{i,1},\;y_{i,1}=j\;if\;\mu_{j-1}<y_{i,1}^{*}<\mu_{j},\;j=0,\cdots J_{1}\\ y_{i,2}^{*}=\beta_{2}X_{i,2}+\varepsilon_{i,2},\;y_{i,2}=k\;if\;\theta_{k-1}<y_{i,2}^{*}<\theta_{k},\;k=0,\cdots K_{2} \end{array}\right.$$

where $y_{i,1}^{*}$ and $y_{i,2}^{*}$ represent potential dependent variables; $y_{i,1}$ and $y_{i,2}$ represent the observed results, that is, the ordinal data (1, 2, 3, 4, 5) and the number of the crashes involved (1, 2, 3, 4, 5) after policy release, respectively; $X_{i,1}$ and $X_{i,2}$ are the vectors of the explanatory variables in the two models; $\beta_{1}$ and $\beta_{2}$ are the column vectors of the correlation coefficients of the explanatory variables in the two models; μ and θ represent the estimated threshold parameters that define $y_{i,1}$ and $y_{i,2}$; $\varepsilon_{i,1}$ and $\varepsilon_{i,2}$ represent the values of the two random error terms of the two models, and are normally distributed with a mean value of 0 and a variance of 1; ρ is the correlation coefficient; i represents the observation sample; and j and k represent helmet usage and crash involvement after policy release, respectively.

The related error term of the cross-equation in the BOP model is given by the following formula:

(2) $$\left[\begin{array}{l} \varepsilon_{i,1}\\ \varepsilon_{i,2} \end{array}\right]\sim N\left(\left[\begin{array}{l} 0\\ 0 \end{array}\right],\left[\begin{array}{cc} 1 & \rho \\ \rho & 1 \end{array}\right]\right)$$

where ρ represents the correlation coefficient between $\varepsilon_{i,1}$ and $\varepsilon_{i,2}$.

Under the assumption of the bivariate normal distribution of the random error term, the joint probability of $y_{i,1}=j$ and $y_{i,2}=k$ can be expressed as follows:

(3) $$\begin{array}{l} P\left(y_{i,1}=j,y_{i,2}=k|X_{i,1},X_{i,2}\right)\\ =\mathrm{Pr}\left(\mu_{j-1}<y_{i,1}^{*}<\mu_{j};\theta_{k-1}<y_{i,2}^{*}<\theta_{k}\right)\\ =\mathrm{Pr}\left(\mu_{j-1}<\beta_{1}X_{i,1}+\varepsilon_{i,1}<\mu_{j};\theta_{k-1}<\beta_{2}X_{i,2}+\varepsilon_{i,2}<\theta_{k}\right)\\ =\mathrm{Pr}\left(\mu_{j-1}-\beta_{1}X_{i,1}<\varepsilon_{i,1}<\mu_{j}-\beta_{1}X_{i,1};\theta_{k-1}-\beta_{2}X_{i,2}<\varepsilon_{i,2}<\theta_{k}-\beta_{2}X_{i,2}\right)\\ =\phi_{2}\left[\left(\mu_{j}-\beta_{1}X_{i,1}\right),\left(\theta_{k}-\beta_{2}X_{i,2}\right),\rho \right]-\phi_{2}\left[\left(\mu_{j-1}-\beta_{1}X_{i,1}\right),\left(\theta_{k}-\beta_{2}X_{i,2}\right),\rho \right]\\ -\phi_{2}\left[\left(\mu_{j}-\beta_{1}X_{i,1}\right),\left(\theta_{k-1}-\beta_{2}X_{i,2}\right),\rho \right]+\phi_{2}\left[\left(\mu_{j-1}-\beta_{1}X_{i,1}\right),\left(\theta_{k-1}-\beta_{2}X_{i,2}\right),\rho \right] \end{array}$$

where $\phi_{2}\left(.\right)$ represents the standard bivariate normal cumulative distribution function.

#### 3.3.2. BOP Model Estimation

The parameters estimated in the BOP model are the threshold, j+k−2; the coefficient vectors, $\beta_{1}$ and $\beta_{2}$; and the correlation coefficient ρ. The parameters can be estimated by maximizing the log-likelihood function, given as follows:

(4) $$LL=\sum_{i=1}^{n}\left(\sum_{j=0}^{J}\sum_{k=0}^{K}\xi_{jk}\left[\begin{array}{l} \begin{array}{l} \phi_{2}\left[\left(\mu_{j}-\beta_{1}X_{i,1}\right),\left(\theta_{k}-\beta_{2}X_{i,2}\right),\rho \right]\\ -\phi_{2}\left[\left(\mu_{j-1}-\beta_{1}X_{i,1}\right),\left(\theta_{k}-\beta_{2}X_{i,2}\right),\rho \right] \end{array}\\ \begin{array}{l} -\phi_{2}\left[\left(\mu_{j}-\beta_{1}X_{i,1}\right),\left(\theta_{k-1}-\beta_{2}X_{i,2}\right),\rho \right]\\ +\phi_{2}\left[\left(\mu_{j-1}-\beta_{1}X_{i,1}\right),\left(\theta_{k-1}-\beta_{2}X_{i,2}\right),\rho \right] \end{array} \end{array}\right]\right)$$

where i=1,2,⋯,n (sample size). If the observed results $y_{i,1}=j$ and $y_{i,2}=k$, then $\xi_{jk}$ is defined as being equal to 1; otherwise, it is 0.

#### 3.3.3. Marginal Effect

After model estimation, the signs of the coefficients associated with the explanatory variables are correlated, which indicates the positive or negative influence of the variable on the result. However, the coefficients can neither quantify the effects of these variables, nor can they intuitively explain them, especially for intermediate categories. To quantify the impact of each type of result, the marginal effects of the related variables in the BOP model are calculated.

The marginal effect of explanatory variable $X_{i,1}$ on $y_{i,1}$ is as follows:

(5) $$\frac{P\left(y_{i,1}=j\right)}{\partial X_{i,1}}=\left[\phi \left(\mu_{j-1}-\beta_{1}X_{i,1}\right)-\phi \left(\mu_{j}-\beta_{1}X_{i,1}\right)\right]\beta_{1}$$

where ϕ(.) is the probability mass function of the standard normal distribution. Similarly, the marginal effect of the explanatory variable $X_{i,2}$ on $y_{i,2}$ is as follows:

(6) $$\frac{P\left(y_{i,2}=k\right)}{\partial X_{i,2}}=\left[\phi \left(\theta_{k-1}-\beta_{2}X_{i,2}\right)-\phi \left(\theta_{k}-\beta_{2}X_{i,2}\right)\right]\beta_{2}$$

### 3.4. Calculation Process and Steps

**Figure 4:**
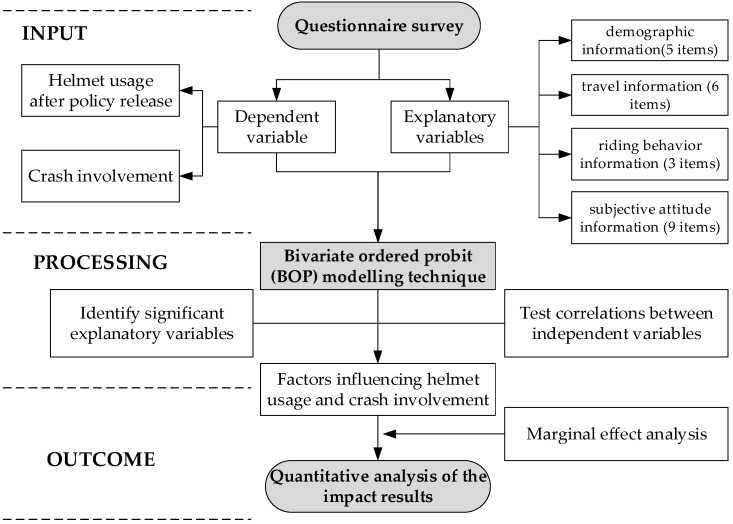
Detailed calculation process and steps.

The process of this study includes three stages: input, processing and outcomes, as shown in Figure 4. In the input part, we take helmet usage after policy release and crash involvement as two dependent variables, and we propose 33 explanatory variables, involving demographic information, travel information, riding behavior information and subjective attitude information, as potential independent variables. We obtained 1048 sets of complete and valid data through questionnaires, which were entered into the bivariate ordered probit (BOP) model for relevant modeling.

After processing, we identify significant explanatory variables in the BOP model as independent variables, and test the correlation between the independent variables. Finally, the significant explanatory variables with weak correlations are output as the independent variables of the model, i.e., the factors affecting helmet usage and crash involvement. Based on this, we calculate the marginal effects in order to quantify and analyze the degree of influence of the respective variables on helmet usage and crash involvement.

The detailed solution steps for the BOP model are as follows,

Step1: Define the explanatory variables as *y*_1_ and *y*_2_, and the individual explanatory variables as *x*_1_, *x*_2_, …, *x_n_*;

Step2: Create two independent probit models;

Step3: Organize the data—we store it in .dat format and import it into STATA software;

Step4: Regression analysis using the BOP model to test the significance of the explanatory variables;

Step5: Request a ρ-value > 0.05, exclude non-significant variables for another regression analysis, and re-calibrate the assessment results;

Step6: In the STATA software, we use the additional command “mfx” to calculate the marginal effect, where the options select dydx for the calculation;

Step7: After the run, the marginal effects of each explanatory variable are analyzed.

## 4. Results and Discussion

### 4.1. Survey Results

**Figure 5:**
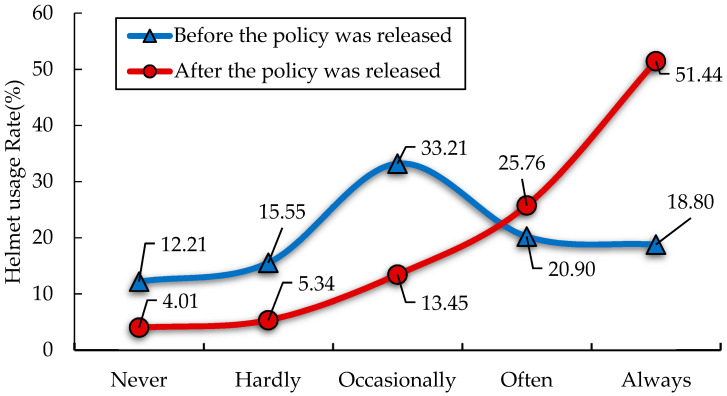
Distribution of helmet usage in Ningbo, China.

Figure 5 shows the wearing of helmets in Ningbo, China, before and after the introduction of the policy. As shown in Figure 5, before the helmet policy was released, the helmet usage rate of e-bike riders in Ningbo was evenly distributed, and the frequency of occasionally wearing a helmet was slightly higher than other frequencies, accounting for 33.21%; the helmet usage rate of e-bike riders in Ningbo showed a significant reduction from five (always) to one (never) on the Likert scale, and the proportions of always and often wearing helmets were as high as 51.44% and 25.76%, respectively. It can be seen from the results that the release of the helmet policy in Ningbo has greatly increased the frequency of helmet usage by e-bike riders. At the same time, through the questionnaire survey, we also found that the riding environment of electric bicycles in Ningbo is relatively safe. Nearly 60% of the respondents said they had never been involved in a cycling crash in Ningbo, China; 18.23% of e-bikes riders had been involved in one cycling crash and only 4.96% had had more than three cycling crashes in Ningbo, China.

### 4.2. Model Estimation

To determine helmet usage and crash involvement, the BOP model was estimated. The explanatory variables and descriptive statistics are shown in Table 1, Table 2, Table 3 and Table 4. The BOP model’s estimation results are shown in Table 5. The BOP model shows a significant correlation between the frequency of helmet usage and crash involvement. Only variables that are significant at the 95% confidence level are included in the final estimation model. According to the relevant data in the table, a higher helmet usage rate can reduce the possibility of crash involvement.

### 4.3. Model Results and Discussion

In the model analysis, we found that 20 variables were not significant in the model, such as gender, age group, education, occupation, travel distance, frequency, etc. As shown in Table 5, the models for helmet usage and crash involvement have 8 and 11 variables, respectively. The goodness of fit of the models of the regression was calculated, and the coefficient of determination R2 = 0.670. The parameters in Table 5 provide the general meaning of the factors contributing to the direction of the results, and Table 6 and Table 7 show the marginal effects of these variables in order to quantify their impacts. The quantitative influence of each factor on the results can be found in Table 6 and Table 7.

According to the estimation results of the BOP model, the evening peak is an important reason for crash involvement (*β* = 0.370), which may be because, at that time, users are in a state of fatigue after a day’s work, which leads to a lack of attention during cycling, thus leading to crash involvement. Our findings are consistent with those of [60,61]. The crash involvement of shoppers is also relatively high (*β* = 0.217), which may be caused by people’s lack of attention while leisure shopping. Similarly, users are also prone to traffic accidents when they pick up their children (*β* = 0.259), which may be caused by illegal nonmotor vehicle cycling, the absence of child safety seats, or parents’ distractions due to communication with their children during cycling. This was confirmed by the findings of [62,63]. In addition, users with higher cycling proficiency (*β* = −0.101) and those who think that the road security level is higher (*β* = −0.105) are less likely to have accidents. This is because the users themselves have higher cycling quality and strong adaptability to road security, and are thus less likely to have accidents than those who are less skilled in cycling and unfamiliar with the environment. The model also shows that users with higher income levels are more likely to experience crash involvement (*β* = 0.096) because they are not skilled at cycling. Moreover, the more severe the helmet policy is (*β* = −0.094), the less likely users are to experience crash involvement, which also reflects that the helmet policy is helpful for reducing the number of traffic accidents, and indicates that the strict management of cycling behavior is helpful for reducing the incidence of accidents. The model estimation results of the safety helmet wearing rate show that users who go out after 19:00 have a higher frequency of helmet usage (*β* = 0.286), which indicates that these users have a stronger awareness of safety protection at night. The results of [63,64] also show that helmet wearing was higher among e-bike riders during the night. Users who use e-bikes to transfer from bus to bus (or subway) wear safety helmets less frequently (*β* = −0.183), which may be due to the tight time constraints of public transport. Users with higher cycling proficiency (*β* = 0.135) and stronger road security (*β* = 0.088) also have a higher probability of wearing safety helmets, which indicates that skilled e-bike riders have relatively higher safety awareness and a higher recognition of environmental safety. Users who had a higher helmet wearing rate before policy release also had a higher helmet wearing rate after policy release (*β* = 0.091), which indicates that users with the habit of wearing safety helmets have higher safety awareness. In addition, people seldom wear safety helmets on sunny days (*β* = 0.233), which may be due to the higher temperature on such days making the wearing of safety helmets stuffier. However, most users who do not want to wear safety helmets feel that it is unnecessary to wear them (*β* = −0.209), and these findings are consistent with those of [64,65], which shows that these people still have a relatively weak awareness of travel safety protection and do not have a good understanding of the protective effects of safety helmets.

The calculation results of the marginal effect show that the occurrence of crash involvement is reduced due to the improvement of cycling proficiency and road security, which is consistent with the estimation results of the BOP model. At the same time, crash involvement is more likely to occur during the evening rush hour, during leisure shopping and when picking up children, which is also described by the BOP model’s estimates. Users who think that helmet policies are more punitive have a marginally lower crash involvement rate. Conversely, the marginal effect of the safety helmet wearing rate shows that the proportion of users going out after 19:00 increases, which indicates that users have relatively strong safety awareness at night, and are more willing to wear safety helmets at night to reduce their possibility of crash involvement. In addition, the marginal effect results show that users with higher cycling proficiency and a strong sense of safety in the road environment are more likely to wear safety helmets, which reflects the importance of cycling technology and road management planning. Before policy release, people who were in the habit of wearing safety helmets had high proficiency in e-bike riding, and those who knew the correct way to wear safety helmets had a higher frequency of wearing safety helmets, which findings are consistent with those of the estimated results of the BOP model.

In addition, the occupancy rate of safety helmets and the registration rate of e-bikes in Ningbo are 88.45% and 93.70%, respectively, which shows that the management of e-bikes in Ningbo is relatively standardized, and that riders generally abide by the related laws and regulations. However, 41.03% of respondents think that the punishment intensity of the helmet policy is appropriate, while only 12.02% and 9.45% of respondents think that the punishment intensity is very heavy or very light, respectively, which indicates that the punishment intensity of the policy is generally approved by users.

Compared with other riders, deliverymen tend to ride on roads more often, and their riding behavior characteristics may be quite different from those of other types of riders. In this survey, 103 questionnaires were filled in by deliverymen, accounting for 9.83%. Among them, most cyclists covered distances of 1~3 km and 3~5 km, accounting for 27.18% and 25.24%, respectively. The most common time for cycling is the evening rush hour, with up to 83 persons choosing it, which is presumed to be due to the large numbers of diners in the evening. Meanwhile, most of these people ride for work and travel, but some deliverymen use cars or other means of transportation to deliver goods, and use electric cars for other travel purposes. Before the implementation of the policy, 31 persons chose “Occasionally” as the most popular choice. After the implementation of the policy, most deliverymen chose to wear helmets, which also reflects the great impact of the policy on the delivery industry.

### 4.4. Correlation Analysis

To explore the association between the dependent variables helmet usage and crash involvement, we have conducted a correlation analysis between the two, as shown in Table 8 and Table 9.

Table 8 shows the results of the descriptive statistics variables; Table 9 shows the results of the correlation analysis. From Table 9, we see that the correlation coefficient between the number of punishments and helmet usage frequency after policy release is −0.493, which indicates a negative correlation, and the absolute value of the correlation coefficient is greater than 0.4, which is a tight correlation. Additionally, at *p* < 0.01, the result is very significant. From Table 9, it can be seen that: with the increase in helmet wearing rate, the accident rate will be greatly reduced.

### 4.5. Measures for Improving Helmet Policy

The results show that the correct and effective wearing of safety helmets can effectively reduce the occurrence of accidents in the process of e-bike riding. However, the dissemination of helmet policies and knowledge of the correct way to wear safety helmets is still limited to a certain extent, and the public’s safety awareness has not been fully popularized. Some citizens in Ningbo are not familiar with the policy, which hinders its promotion and regulation. Therefore, a variety of other communication methods can be adopted to improve the communication power of the Ningbo helmet policy, such as real-time broadcasting at intersections, communication by public transportation media, school and enterprise websites, or official accounts.

According to the questionnaire, many cyclists are reluctant to wear helmets because e-bike riders think they are uncomfortable to wear, and they also feel that wearing a helmet obscures their vision when riding. It is suggested that the safety helmet manufacturers can improve and adjust the performance of safety helmets according to these two points. Hot weather and rainy days are also cited as reasons for cyclists not to wear safety helmets. It is suggested that the traffic department should reasonably increase the number of road sunshades to improve the travel experience of e-bike riders. At the same time, it can be found that some cyclists who think wearing helmets is unnecessary have a lower helmet wearing rate than other cyclists, so we suggest that safety helmet manufacturers and government safety management departments should vigorously promote the importance of wearing helmets.

In addition, cycling accidents are more likely to occur during the evening rush hour and when cyclists are leisure shopping. It is suggested that traffic management departments strengthen traffic control during the evening rush hour and around busy commercial cities.

Meanwhile, in view of the outbreak of the coronavirus disease (COVID-19) in 2019, preventing the epidemic has been the primary task for riders. Helmet manufacturers should respond to this by manufacturing helmets with protective functions similar to masks. Additionally, the government should also provide subsidies to riders to maintain a high level of helmet ownership and avoid cross infection.

This study shows that there is a negative correlation between the popularity and implementation of a helmet policy and the accident rate of e-bikes in Ningbo. Therefore, it is necessary to continue to spread and promote the implementation of helmet policy and constantly revise and improve the relevant content of such policy, so that the safety of cyclists can be better guaranteed.

## 5. Conclusions

Although relevant laws and regulations on helmet wearing for e-bike riders have been issued in various places, there are fewer studies on the factors affecting helmet usage and crash involvement caused by the release of helmet policies. This study uses the BOP model to identify the factors that affect helmet usage and crash involvement. The questionnaire survey method has been used to collect data from Ningbo. The survey results show that most users have a high degree of compliance with the policy and that helmet usage has increased significantly after the policy release. However, the correct way to wear a helmet, the relevant regulations of the helmet policy and the awareness of cycling safety are still relatively low among riders. A total of 1048 valid samples have been used to develop the BOP model. In addition, this study provides several measures for policy improvements based on the model’s results.

With the rapid development of transportation in China, electric bicycles have become one of the main ways for people to travel in their daily lives. With the improvement of people’s economic living standards, the per capita ownership and utilization rate of electric bicycles have been significantly improved. Therefore, it is urgent to formulate and improve laws and regulations that can effectively and reasonably regulate electric bicycle riding and strengthen the safety of citizens. The results of this study can provide useful information and guidance for improving the new helmet policy in Ningbo.

The results show that the correct and effective usage of safety helmet can reduce the occurrence of crash involvement in the process of e-bike riding. However, the new helmet policy and the dissemination of the correct way to wear a helmet are still limited to a certain extent, and the public’s safety awareness has not been fully popularized. Some citizens in Ningbo are not familiar with the new policy and do not understand it, which hinders its promotion and regulation. Therefore, a variety of other communication methods can be adopted to improve the communication power of the new helmet policy in Ningbo, such as real-time broadcast at intersections, public transport media, school and enterprise websites, or official accounts. This study shows that there is a negative correlation between the popularity and implementation of the new helmet policy and the accident rate of e-bike riders in Ningbo. Therefore, we should continue to spread and promote the implementation of the new helmet policy, and constantly improve and modify the relevant content of the new helmet policy in the future to better guarantee the travel safety of cyclists.

In China, e-bike usage is at a high level in many large cities. However, the safety level of e-bikes in some cities is not satisfactory. The high accident rate of electric bicycles is a key problem that hinders the sustainable development of this mode of transportation. A better understanding of the factors that influence helmet-wearing frequency and e-bike cycling accident rates could provide solutions to this problem. Based on the results of this study, several useful improvements to reduce the accident rate of electric bicycles are discussed. Future studies could also help persuade drivers to wear e-bike safety helmets more efficiently and safely.

However, several limitations still exist in our work, and future work will focus on two aspects. For example, (a) the sample data investigated in this study are all from Ningbo city, ignoring the influence of regional conditions and different policy conditions on cyclists’ behavior. (b) It is extremely important for such work to link with COVID-19, which has grounded a paradigm shift; our study was conducted before the pandemic, but up to now, the situation has been rapidly changing. Hence, in future studies, the data collection needs to take into account the impact of the epidemic, and new research needs to fit to post-COVID realities. Additionally, the effective sample size of this paper is 1048. If we want to understand the impact of different geographical environments and different helmet policies on the cycling behavior of e-bike users, then the sample size of the investigation should be further expanded to optimize the constructed BOP model and improve the analysis of the impact of various factors influencing the cycling behavior of e-bike riders.

## Figures and Tables

**Table 1:** Descriptive statistics for demographic information.

Variable	Description	Symbol	Frequency	Proportion (%)
Gender	Male	1	613	58.49
	Female	2	435	41.51
Age group	Young people (12–29)	1	404	38.55
	Middle-aged people (30–49)	2	530	50.57
	Old people (50–70)	3	114	10.88
Education	Below junior high school degree	1	78	7.44
	High school and junior high school degree	2	295	28.15
	Bachelor degree	3	572	54.58
	Master degree and above	4	103	9.83
Occupation	Student	1	120	11.45
	Company/corporate employee	2	316	30.15
	Housewife	3	41	3.91
	Private owners	4	202	19.27
	Freelancer	5	204	19.47
	Deliveryman	6	103	9.83
	Retirement	7	44	4.20
	Other	8	18	1.72
Monthly income	<¥2000	1	172	16.41
	¥2000~5000	2	386	36.83
	¥5000~8000	3	310	29.58
	>¥8000	4	180	17.18

**Table 2:** Descriptive statistics for travel information.

Variable	Description	Symbol	Frequency	Proportion (%)
Travel distance	<1 km	1	186	17.75
	1~3 km	2	340	32.44
	3~5 km	3	287	27.39
	5~7 km	4	140	13.36
	>7 km	5	95	9.06
Frequency	Almost never	1	68	6.49
	Occasionally	2	192	18.32
	Often	3	328	31.30
	Always	4	224	21.37
	Everyday	5	236	22.52
Time period (multiple choice)	Morning peak	1	742	70.80
	Evening peak	2	748	71.37
	Noon	3	287	27.39
	After 19 o’clock	4	170	16.22
	Other	5	138	13.17
Purpose (multiple choice)	Commute to get off work/school	1	512	48.85
	Bus/subway transfer	2	237	22.61
	Work trip	3	512	48.85
	Shopping	4	482	45.99
	Pick up children	5	342	32.63
	Other	6	142	13.55
Whether the electric bike is licensed	Yes	1	982	93.70
	No	0	66	6.30
Whether the rider has a helmet	Yes	1	927	88.45
	No	0	121	11.55

**Table 3:** Descriptive statistics for cycling behavior information.

Variable	Description	Symbol	Frequency	Proportion (%)
Helmet usage frequency before policy release	Never	1	128	12.21
	Almost never	2	163	15.55
	Occasionally	3	348	33.21
	Often	4	212	20.23
	Always	5	197	18.80
Helmet usage frequency after policy release	Never	1	42	4.01
	Almost never	2	56	5.34
	Occasionally	3	141	13.45
	Often	4	270	25.76
	Always	5	539	51.44
Number of crash involvements	Yet to happen	1	606	57.82
	1 time	2	191	18.23
	2 times	3	114	10.88
	3 times	4	85	8.11
	More than 3 times	5	52	4.96
Whether the rider wears a helmet at the time of survey	Yes	1	813	77.58
	No	0	235	22.42
Instances of punishment when a helmet is not worn in cycling behavior	None	1	606	57.82
	1 time	2	191	18.23
	2 times	3	114	10.88
	3 times	4	85	8.11
	More than 3 times	5	52	4.96

**Table 4:** Descriptive statistics for subjective attitude information.

Variable	Description	Symbol	Frequency	Proportion (%)
Degree of understanding that WHO points out that helmets can reduce the risk of death and injury	Totally no idea	1	248	23.66
	Understand	2	473	45.13
	Know exactly	3	327	31.20
Whether they know the policy	Yes	1	778	74.24
	No	0	270	25.76
Whether they are safe after wearing a helmet	Yes	1	849	81.01
	No	0	199	18.99
Cycling proficiency	Poor	1	92	8.78
	General	2	158	15.08
	Better	3	232	22.14
	Good	4	319	30.44
	Very good	5	247	23.57
Road security	Poor	1	87	8.30
	General	2	132	12.60
	Safer	3	340	32.44
	Safety	4	333	31.77
	Very safe	5	156	14.89
Punishment degree	Very light	1	99	9.45
	Lighter	2	114	10.88
	Moderate	3	430	41.03
	Heavier	4	279	26.62
	Serious	5	126	12.02
Reasons for reluctantly wearing a helmet (multiple choices)	Feels unnecessary	1	161	15.36
	Uncomfortable to wear	2	542	51.72
	Price is too high	3	232	22.14
	Feel unsightly	4	394	37.60
	Block the line of sight after wearing	5	511	48.76
	Too troublesome to wear	6	390	37.21
Weather when they reluctantly wear a helmet (multiple choice)	Rain	1	588	56.11
	Hot day	2	596	56.87
	Cloudy day	3	318	30.34
	Sunny day	4	292	27.86
	No	5	151	14.41
Helmet wearing proficiency	Totally no idea	1	112	10.69
	Probably know	2	479	45.71
	Know exactly	3	457	43.61

**Table 5:** Estimated results of the BOP model.

Variable	Number of Crashes			Helmet Usage after Policy Release		
	β	S.E.	*p*-Value	β	S.E.	*p*-Value
Use time period (evening peak)	0.370	0.115	0.001 *	-	-	-
Use time period (after 19 o’clock)	-	-	-	0.286	0.118	0.015
Purpose (bus/subway transfer)	-	-	-	−0.183	0.092	0.046
Purpose (shopping)	0.217	0.087	0.013 *	-	-	-
Purpose (pick up children)	0.259	0.087	0.003 *	-	-	-
Purpose (other)	0.239	0.113	0.034	-	-	-
Helmet usage Before policy release	-	-	-	0.091	0.029	0.002 *
Weather when they reluctantly wear a helmet (sunny day)	-	-	-	−0.233	0.091	0.011
Cycling proficiency	−0.105	0.040	0.009 *	0.135	0.038	0.000 *
Road security	−0.101	0.040	0.011	0.088	0.038	0.021
Number of punishments when a helmet is not worn during cycling	0.156	0.035	0.000 *	-	-	-
Punishment degree	−0.094	0.039	0.017	-	-	-
Reasons to reluctantly wear a helmet (feels unnecessary)	-	-	-	−0.209	0.107	0.050
Whether wearing a helmet	−0.898	0.120	0.000 *	0.793	0.117	0.000 *
Helmet wearing proficiency	−0.149	0.056	0.008 *	-	-	-
Monthly income	0.096	0.048	0.046	-	-	-
Number of observations	1048					

* represents *p*-Value < 0.01, which indicates that the corresponding variable is very significant.

**Table 6:** Marginal effect of the crash involvement BOP model.

Crash involvement (*n*)	*n* = 0	*n* = 2	*n* > 3
Use time period (evening peak)	−0.172	0.057	0.022
Purpose (shopping)	−0.070	0.023	0.009
Purpose (pick up children)	−0.097	0.032	0.012
Purpose (other)	−0.086	0.029	0.011
Cycling proficiency	0.038	−0.013	−0.005
Road security	0.037	−0.012	−0.005
Number of punishments when a helmet is not worn in cycling behavior	−0.067	0.022	0.009
Punishment degree	0.040	−0.013	−0.005
Whether wearing a helmet	0.348	−0.116	−0.045
Helmet wearing proficiency	0.065	−0.022	−0.008
Monthly income	−0.051	0.017	0.007

**Table 7:** Marginal effect of the helmet usage BOP model after policy release.

Helmet Usage after Policy Release	Never	Occasionally	Always
Use time period (after 19 o’clock)	−0.013	−0.045	0.109
Purpose (bus/subway transfer)	0.012	0.040	−0.098
Helmet usage before policy release	−0.004	−0.015	0.036
Weather when they reluctantly wear a helmet (sunny day)	0.011	0.039	−0.096
Cycling proficiency	−0.007	−0.023	0.056
Road security	−0.005	−0.016	0.039
Reasons why they reluctantly wear a helmet (feels unnecessary)	0.008	0.027	−0.065
Whether they wear a helmet	−0.035	−0.121	0.295

**Table 8:** Descriptive statistics results.

Parameters	Mean	Std. Deviation	N
Number of punishments	1.842	1.1976	1048
Helmet usage frequency after policy release	4.153	1.0960	1048

**Table 9:** Correlations results.

Parameters		Number of Punishments	Helmet Usage Frequency after Policy Release
Number of punishments	Pearson Correlation	1	−0.493 **
	Sig. (2-tailed)		0.000
	Sum of Squares and Cross-Products	1501.706	−677.656
	Covariance	1.434	−0.647
	*N*	1048	1048
Helmet usage frequency after policy release	Pearson Correlation	−0.493 **	1
	Sig. (2-tailed)	0.000	
	Sum of Squares and Cross-Products	−677.656	1257.573
	Covariance	−0.647	1.201
	*N*	1048	1048

** Correlation is significant at the 0.01 level (2-tailed).

## Data Availability

The raw data supporting the conclusions of this article will be made available by the authors upon reasonable request.
